# Sub-concussive head impacts from heading footballs do not acutely alter brain excitability as compared to a control group

**DOI:** 10.1371/journal.pone.0306560

**Published:** 2024-08-01

**Authors:** Raphael Hamel, Baptiste Maxime Waltzing, Tom Massey, James Blenkinsop, Leah McConnell, Kieran Osborne, Karamo Sesay, Finn Stoneman, Adam Carter, Hajar Maaroufi, Ned Jenkinson

**Affiliations:** 1 School of Sports, Exercise, and Rehabilitation Sciences, University of Birmingham, Birmingham, United Kingdom; 2 Institute of Neurosciences, UC Louvain, Bruxelles, Belgium; Monash University School of Public Health and Preventive Medicine, AUSTRALIA

## Abstract

**Background:**

Repeated sub-concussive head impacts are a growing brain health concern, but their possible biomarkers remain elusive. One impediment is the lack of a randomised controlled human experimental model to study their effects on the human brain.

**Objectives:**

This work had two objectives. The first one was to provide a randomised controlled human experimental model to study the acute effects of head impacts on brain functions. To achieve this, this work’s second objective was to investigate if head impacts from heading footballs acutely alter brain excitability by increasing corticospinal inhibition as compared to a control group.

**Methods:**

In practised and unpractised young healthy adults, transcranial magnetic stimulation was used to assess corticospinal silent period (CSP) duration and corticospinal excitability (CSE) before and immediately after performing headings by returning 20 hand-thrown balls directed to the head (Headings; n = 30) or the dominant foot (Control; n = 30). Moreover, the Rivermead Post-Concussion Questionnaire (RPQ) was used to assess the symptoms of head impacts. Head acceleration was also assessed in subgroups of participants.

**Results:**

The intervention lengthened CSP duration in both the Headings (6.4 ± 7.5%) and Control groups (4.6 ± 2.6%), with no difference in lengthening between the two groups. Moreover, CSE was not altered by the intervention and did not differ between groups. However, performing headings increased headaches and dizziness symptoms and resulted in greater head acceleration upon each football throw (12.5 ± 1.9g) as compared to the control intervention (5.5 ± 1.3g).

**Conclusions:**

The results suggest that head impacts from football headings do not acutely alter brain excitability as compared to a control intervention. However, the results also suggest that the present protocol can be used as an experimental model to investigate the acute effects of head impacts on the human brain.

## Introduction

Repeated sub-concussive head impacts are increasingly recognised to have detrimental effects on the brain [1]. For instance, repeated sub-concussive head impacts negatively alter white matter tract integrity, reduce cortical thickness, and impair cognitive functions in both animals [2, 3] and humans [4, 5]. Although sub-concussive head impacts can have various origins, such as falls and automobile collisions, contact sports constitute an increasingly important vector of head impacts [6]. For instance, a history of sport-related head impacts increases the risk of developing neurodegenerative diseases and/or cognitive impairments in American [7–9] as well as European football players [10–12]. Given that head impacts are modifiable risk factors [13], and that the socioeconomic burden of neurodegenerative diseases is increasing worldwide [14], there is a pressing need to characterize the full effects of sub-concussive head impacts on brain health using randomised controlled experimental settings [15, 16]. In this light, one of this work’s objectives was to provide a randomised controlled human experimental model to study the effects of sub-concussive head impacts on the brain. Similar to work on concussions [17], such systematic investigations could ultimately lead to recommendations to attenuate (or manage) the detrimental effects of head impacts on the brain.

One crucial feature of brain health is the maintenance of a balance between brain excitation and inhibition, as any imbalances in brain excitability are associated with a range of psychiatric symptoms [18–20]. Interestingly, converging work indicates that head impacts deteriorate brain health by–amongst other mechanisms–altering brain excitability [1, 21, 22]. Importantly, changes in brain excitability can be readily assessed in humans [23], offering a unique opportunity to obtain insights into the effects of head impacts on brain health [1]. For instance, Di Virgilio et al. (2016; ref [24]) used a battery of cognitive tests and transcranial magnetic stimulation (TMS) to assess how head impacts from heading 20 footballs over 10 minutes alter cognitive functions and brain excitability. Their results showed that the football headings acutely impaired working memory and declarative learning performances. Moreover, the results also showed that the headings acutely increased the duration of the corticospinal silent period (CSP), a TMS-derived change in brain excitability believed to reflect increased gamma-aminobutyric acid (GABA)ergic-mediated inhibition in the corticospinal tract [23]. Overall, these findings suggest that sub-concussive head impacts alter brain excitability by increasing inhibition (for similar results, see refs [25, 26]), in turn impairing cognitive functions. Not unexpectedly, this work attracted considerable mediatic attention, notably in the form of a documentary (see ref [27]). However, it remains unclear if the brain excitability changes were caused by the football headings because the results were crucially not compared to a control group that did not experience head impacts. Despite this limitation, the protocol used by the authors constitutes a promising experimental model because it allows to experimentally induce head impacts in a controlled environment. Furthermore, changes in CSP duration constitute a promising biomarker of GABAergic inhibition to investigate changes in brain excitability, as measuring CSP is non-invasive, can be easily and quickly measured, and its utility to evaluate changes in brain excitability is supported by work on both concussive (reviewed in ref [22]) and sub-concussive head impacts (reviewed in ref [1]). Overall, this evidence suggests that measuring changes in CSP duration before and after performing football headings constitutes a promising human experimental model to characterise the effects of head impacts on brain excitability.

Based on this background evidence, this work’s primary objective was to substantiate the possibility that football headings can be used as a randomised controlled experimental model to study the acute effects of head impacts on brain functions. To achieve this, this work’s second objective was to build on the results from Di Virgilio et al. (2016; ref [24]) using a randomised controlled trial–by adding an appropriate control group–to ascertain that the changes in CSP duration originate from head impacts. Namely, TMS was used to assess changes in CSP duration before and immediately after groups of practised and unpractised young healthy adults performed (Headings; n = 30), or not (Control; n = 30), 20 football headings. It was hypothesised that CSP would acutely lengthen in response to the head impacts (as in ref [24]) *and* as compared to the control group [25, 26]. In addition, the Rivermead Post-Concussion Questionnaire was used to evaluate head impact symptoms before and after the intervention. Head accelerometer data were also recorded in subgroups of participants from the Headings (n = 10) and Control groups (n = 10). It was hypothesised that the football headings would increase generic head impact symptoms, such as headaches and dizziness, and manifest as greater head acceleration as compared to the control intervention.

## Methods

### Participants

Sixty physically and neurologically healthy young adults took part in this study. All participants reported having no history of concussion in the previous 5 years. Based on prior work [28], an absence of concussion history would ensure the integrity of glutamatergic and GABAergic neurotransmission within the motor cortex. This is important for the present study, as changes in glutamatergic and GABAergic activity could importantly confound the assessment of CSP duration and CSE [23], and, consequently, brain excitability. To enhance the sample’s ecological validity, participants with or without expertise (i.e., practised and unpractised) playing in European football were recruited. It was reasoned that the acute effects of sub-concussive head impacts on the brain’s excitability should be apparent regardless of individuals’ expertise so that football headings can be employed as a valid experimental model in humans, including experienced and non-experienced football players as well as non-football players.

The participants were randomly allocated to a group that experienced head impacts (Headings; n = 30) and a group that did not (Control; n = 30). See Table 2 for further details on the groups’ characteristics. Participants were screened for TMS contraindications [29], the major contraindications being the presence of metal particles/objects in the region of the head and the presence of a personal history of epileptic seizures or convulsions. The study was approved by the local research ethics committee of the University of Birmingham (project # ERN-182077AP10). All participants provided written informed consent before their participation. The recruitment period for this study extended from January 20^th^ 2023 to March 20^th^ 2023.

The number of participants was based on a sample size analysis using G*Power (v.3.1.9.4). Namely, the smallest effect size of interest for the context of this study was a Cohen’s d of 0.8 (large effect size). Assuming the use of an independent t-test (to evaluate differences between Headings and Control), 80% statistical power, and a significance threshold of 0.05, the results of the analysis revealed that two groups of 26 participants are required. To ensure a statistical power greater than 80%, two groups of 30 participants were thus recruited. Furthermore, a post-hoc achieved power analysis using G*Power [30] was conducted to determine if the statistical power achieved by this study would be enough to replicate the acute lengthening in CSP duration from Di Virgilio et al. (2016; ref [24]), which reported an average CSP duration increase of 5.4 ± 4.8% (mean ± SD). Converting this within-subject mean (± SD) increase to a Cohen’s dz value using G*Power resulted in an effect size of 1.125 (above-large effect size). Assuming this effect size, a significance threshold of 0.05, and a sample size of 30 participants, the achieved power in the present study was 100%. Overall, these results suggest that two groups of 30 participants are sufficient to detect meaningful differences between the Headings and Control groups and replicate the results from Di Virgilio et al. (2016; ref [24]).

### Study protocol

**Fig 1:**
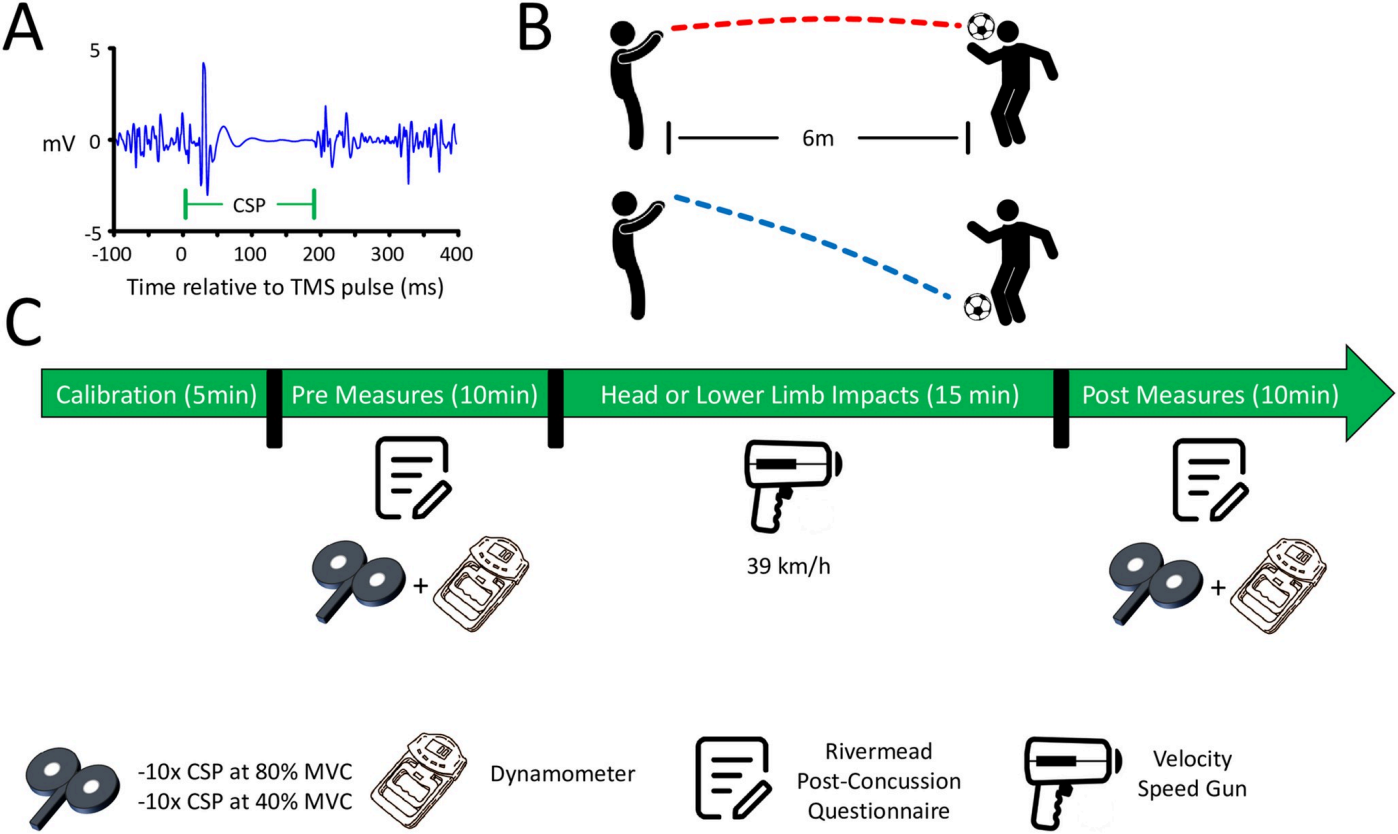
Overview of the protocol. **(A)** *Visual depiction of a corticospinal silent period (CSP)*. A silent period in the surface electromyography (EMG) follows the delivery of a TMS pulse when participants are voluntarily contracting hand muscles. The CSP duration is thought to reflect the extent of GABAergic inhibition in the corticospinal tract [23]. **(B)** *Visual depiction of the Headings and Control groups*. The ball was manually thrown from a 6m distance at a set speed of 39 km/h (measured using a velocity speed gun on every throw). The ball was thrown once every 30 sec for a total of 20 times. Participants either headed the ball (Headings; upper panel) or controlled the ball using their dominant lower limb (Control; lower panel), therefore preventing any head impacts. **(C)** *Timeline of the protocol*. First, participants filled out the Rivermead Post-Concussion Questionnaire and a total of 20 valid CSP trials were collected (10 trials at 80% of the maximum voluntary contraction (MVC) and 10 trials at 40% MVC). Subsequently, participants performed the headings or lower limb ball contacts. Finally, the Rivermead Post-Concussion Questionnaire was filled again and a total of 20 valid CSP trials were collected a second time (10 trials at 80% MVC and 10 trials at 40% MVC). The measures were collected in this specific order for every participant.

An overview of the study protocol is provided in Fig 1. The CSP (Fig 1A), the CSE, and the Rivermead Post-Concussion Questionnaire data were measured before (Pre Measures) and after (Post Measures; Fig 1C) participants either headed the ball or used their dominant lower-limb to control the ball (Fig 1B). In both groups, participants experienced 20 throws [31], at a rate of 1 throw every 30 seconds (similar average throw rate as in Di Virgilio et al., 2016; ref [24]). Trials where participants missed the ball were performed again to ensure that a total of 20 contacts with the ball were experienced. Participants of the Headings group were instructed to head the football to redirect it perpendicularly in the direction of their right-hand side (rotational headers; as in Di Virgilio et al., 2016; ref [24]). Participants of the Control group were instructed to use their dominant lower limb (preferably the foot) to control the ball. Participants of both groups wore a swimming cap during these procedures. This method of control was used to prevent head impacts and match the level of exercise performed between the two groups. All parts of the procedures involving football throws were performed outdoors.

The ball consisted of a standard football (400g, 70 cm circumference; 8 psi) and was manually thrown by an experienced (> 10 years) football player. The ball was thrown from a 6m distance [24]. The ball was either thrown towards the head (Headings) or dominant lower limbs (aiming at the foot; Control). The rationale for manually throwing the ball was to best approximate practice and game conditions, as an effort to enhance ecological validity. Manually throwing the ball deviates from the protocol in Di Virgilio et al. (2016; ref [24]), but aligns with other studies (see ref [31]). The ball was thrown at an average speed of 39km/h [24], which was measured upon each throw with a hand-held Velocity Speed Gun (Bushnell, model 101911). The measured ball speed for the Headings and Control groups was 39.2 ± 0.2 km/h and 39.1 ± 0.2 km/h (mean ± 95% CIs), respectively, confirming that the 39km/h target was successfully achieved. Globally, 40 minutes were required to complete the present protocol (Fig 1C).

Data from a tri-axial WITMOTION wireless accelerometer (model BWT61CL; 100 Hz sampling rate) was recorded from subgroups of 10 participants from the complete Headings (n = 30) and Control groups (n = 30). Specifically, accelerometer data were recorded for three reasons. First, it was to confirm that peak head acceleration upon performing headings would replicate the values reported by Di Virgilio et al. (2016; ref [24]), which were 13.1 ± 1.9g (see ref [31] for similar results). Second, it was to substantiate that peak head acceleration was greater in the Headings than in the Control group, as the control intervention (i.e., moving the lower limb to control the football) was hypothesised to indirectly cause head movements as well. Third, it was to estimate the extent of the head acceleration experienced in the Control group, as it remains unclear if head acceleration–without direct head impacts–can also result in brain injuries [32–34]. This feature is important to investigate, as the head acceleration resulting from head impacts in contact sports is rarely compared to control interventions [35]. Here, before the football throws, the accelerometer was placed under the swimming cap and secured with an adjustable strap to the head over the occipital bone. The accelerometer was removed after the 20 throws were completed.

### Rivermead Post-Concussion Questionnaire

To confirm the headings footballs induced subjective symptoms of head impacts in the Headings but not the Control group, the Rivermead Post-Concussion Questionnaire (RPQ; ref [36]) was administered before and after performing the intervention. The RPQ consists of 16 items (see Table 2 for a list), each evaluated using the following scores: 0 (not experienced at all), 1 (no more of a problem), 2 (a mild problem), 3 (a moderate problem), and 4 (a severe problem). Conceivably, some RPQ items appear to specifically evaluate the symptoms of concussive head impacts (e.g., feeling frustrated, sleep disturbance), arguably rendering these items ill-suited to evaluate symptoms of sub-concussive head impacts. However, a few RPQ items appear well-suited to evaluate some generic symptoms of *sub-concussive* head impacts (e.g., headaches, feeling of dizziness), arguably because some symptoms assessed by the RPQ are not specific to *concussive* head impacts. In the absence of a validated questionnaire specifically developed for assessing the acute symptoms of sub-concussive head impacts, it was reasoned that the RPQ would be sensitive enough to do so in the present study. The results provide post-hoc support for this contention.

### TMS, EMG, and dynamometer

The following procedures were performed indoors, in a temperature-controlled environment. TMS was delivered using a 70mm figure-of-eight D-Alpha flat coil connected to a Magstim 200^2^ stimulator (Magstim, Whitland, UK), which was positioned at a 45° orientation along the posterior-anterior axis (coil’s handle pointing towards the occiput). TMS pulses were delivered to the cortical representation of the first dorsal interosseus (FDI) muscle of the left M1 [26]. Rather than stimulating the rectus femoris (as in Di Virgilio et al., 2016; ref [24]), the FDI was targeted because its cortical representation is superficial [37], can be reliably stimulated [38] and has a low activation threshold [37]. This makes the FDI an ideal target to reliably assess corticospinal inhibition whilst minimising discomfort, offering a convenient way of measuring CSP duration. As justified below, this allowed to record 10 CSP trials, which optimised the reliability with which CSP duration can be estimated [39]. It should be noted that this deviates from Di Virgilio et al. (2016; ref [24]), which recorded 3 CSP trials from the rectus femoris’ cortical representation. Here, the surface electromyography (EMG) data of the FDI muscle of the right hand were recorded using bipolar electrodes connected to a Delsys ^®^ Bagnoli system, itself connected to a Power 1402 data acquisition interface (Cambridge Electronic Design ^®^). The data were digitised at 10,000 Hz for 500ms epochs (100ms pre-trigger time; bandpass filtered between 20 and 450 Hz) and recorded using Signal (v6.05; Cambridge Electronic Design ^®^). The reference electrode was located on the proximal portion of the right ulnar bone.

Participants wore a rubber swimming cap, which was used to mark the location of the motor hotspot (as in the methods of ref [40]), defined as the cortical location where motor-evoked potentials (MEPs) in the FDI were reliably induced using suprathreshold TMS pulses. Vertical lines were additionally drawn at the junction of participants’ skin and swimming cap, to ensure that the swimming cap did not move when performing the headings. Once the motor hotspot was marked on the swimming cap, the stimulator intensity was adjusted to obtain MEPs of ±1mV at rest (hereafter referred to as test stimulus intensity). Specifically, this was done before participants performed voluntary muscle contractions (i.e., at rest), to ensure that TMS induced similar baseline MEP amplitudes for each participant. It was expected that MEP amplitudes would increase above the 1mV target (to ~ 5mV; see Results) whilst participants executed controlled and normalised muscle contractions (see below). Once determined for a given participant, the test stimulus intensity was kept constant throughout the experiment. For all participants (n = 60), the test stimulus intensity was 55 ± 2% (mean ± 95% CIs).

A total of 10 valid CSP trials per level of maximum voluntary contraction (MVC; see below) for both Pre and Post Measures were recorded. This number of trials was chosen because measuring more than 10 CSP trials does not further enhance the reliability of CSP duration estimation [39]. To induce CSP, participants squeezed a hand dynamometer (Kuptone ^®^) at 80% and 40% of their MVC (defined as the greatest force generated out of three trials). Although arguably non-specific to the FDI muscle, the use of a dynamometer was to provide an effective, inexpensive, and accessible means to induce controlled voluntary contractions of hand muscles for on-the-field and experimental settings. Participants were instructed to reach and maintain a contraction at their individualised 80% and 40% MVC targets for ~3sec, allowing sufficient time for the experimenter to deliver a single TMS pulse upon reaching those targets. Approximately 6 seconds separated each TSM pulse. The peak generated force (in kg) was assessed for each CSP trial. The results revealed that all participants (n = 60) exerted peak forces that represented 80 ± 0.1% and 43 ± 0.1% of their MVC (mean ± 95% CIs), confirming that the 80% and 40% targets were achieved. Here, a target of 100% MVC was not used (unlike Di Virgilio et al., 2016; ref [24]), as it generated important fatigue and limited the number of CSP trials that could be recorded. Rather, CSP trials were collected at 80% and 40% MVC to increase the number of CSP trials and determine if similar CSP lengthening could be observed at different MVC (as supported by ref [41]). The latter case being, it would suggest that a lengthening of CSP duration in response to head or lower limb impacts could be observed using low MVC, making it convenient to collect multiple CSP trials in on-the-field and experimental settings.

### Dependent variables

The primary dependent variables were CSP duration and CSE. Using a custom-designed algorithm in MATLAB (R2022b, Mathworks ©), CSP duration was measured as the time difference (in ms) between the delivery of the TMS pulse and the return of the EMG of voluntary muscle activity (CSP offset). Specifically, the CSP offset was determined as the moment when the standard deviation (SD) of a 2.5ms sliding window exceeded 50% of the SD of the EMG background activity, calculated over the 100ms that preceded TMS pulse delivery, for at least 5ms (similar to refs [42, 43]). The EMG data were not rectified. To normalise the CSP duration, the average CSP data (in ms) from the Post Measures were divided by the average CSP data (in ms) from the Pre Measures, resulting in a percent (%) change. This was done separately for each level of Contraction Levels (80%, 40% MVC). To investigate the effects of sub-concussive head impacts on CSE (see ref [44]), peak-to-peak MEP amplitude was assessed (in mV) for each CSP trial included in the analyses. This was done separately for each level of Contraction Levels (80%, 40% MVC). It should be noted that MEP amplitude was not measured at rest but when participants performed voluntary muscle contractions.

The secondary dependent variables were the RPQ scores and the accelerometer data. Namely, the RPQ scores (0 to 4) were averaged across groups for each item (see Table 2). Concerning the accelerometer data, peak linear acceleration was first calculated (in g) and a threshold of 10g was used to determine the occurrence of head impacts [35]. Then, to approximate the overall head impacts experienced over the 10-minute intervention, the peak linear acceleration of all head impact occurrences exceeding 10g was summed and divided by the total number of throws experienced (i.e., 20). This was done separately for each participant of the Headings and Control subgroups.

### Statistical analyses

To analyse the results, mixed ANOVAs were used. The within-subject factors were Measures (Pre, Post) and Contraction Levels (80% MVC, 40% MVC). The between-subject factor was Groups (Headings, Control). If the data violated the assumptions of sphericity (*p* < 0.05, Mauchly test), the Greenhouse-Geiser correction was applied. If data deviated from normality (*p* < 0.05; Shapiro-Wilk test) upon pairwise comparisons, non-parametric pairwise comparisons were conducted (Wilcoxon rank test rather than dependent t-test for within-subject comparisons; U Mann-Whitney test rather than independent t-test for between-subject comparisons). The Benjamini-Hochberg (1995; ref [45]) correction was used to control for inflated type 1 error upon multiple comparisons. The statistical significance threshold was set at 0.05. All the descriptive statistics reported in this work represent the mean ± 95% CIs. The open-access software JAMOVI was used to conduct the statistical analyses.

## Results

### CSP duration lengthened in both the Headings and Control groups

**Fig 2:**
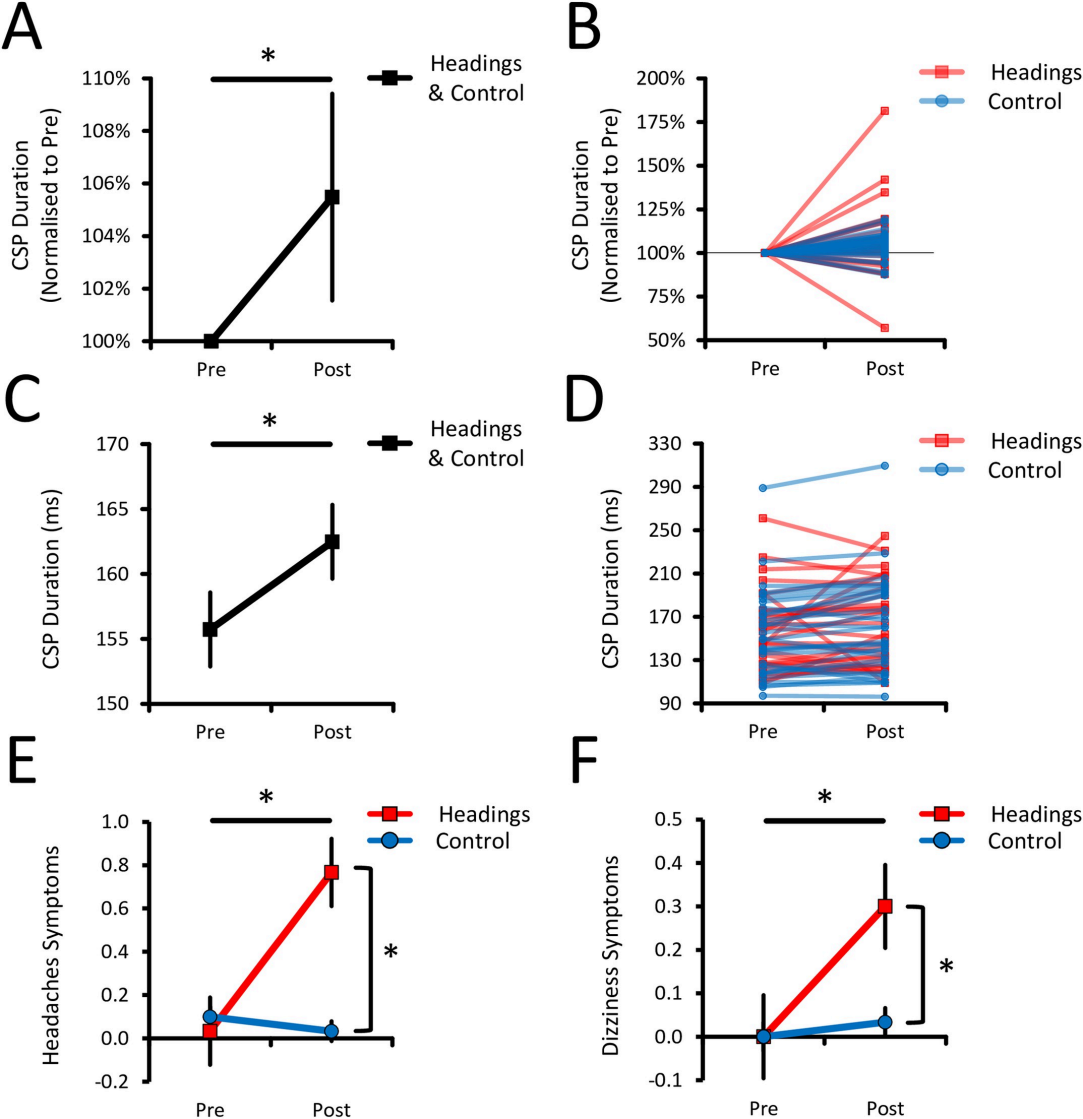
CSP and RPQ results. **(A)** Mean change and **(B)** individual data of the normalised CSP duration, depicted as a percentage change from the Pre Measures. These results show that CSP duration lengthened similarly in both groups, suggesting that corticospinal inhibition increased regardless of whether head impacts from football headings were experienced. **(C)** Mean change and **(D)** individual data of the (non-normalised) CSP duration, depicted as values in milliseconds. This confirms that the results in **(A)** and **(B)** are not a by-product of normalising the CSP duration. **(E)** Headaches and **(F)** Dizziness symptoms, as evaluated with the RPQ. The Headings group showed greater headaches and dizziness symptoms after headings were performed as compared to the Control group. This indicates that symptoms of head impacts were experienced in the Headings, but not in the Control group. For panels (A), (C), (E), and (F), the mean ± 95% CIs are shown. Asterisks (*) denote significant differences.

Concerning the normalised CSP duration data (Fig 2A and 2B), the results revealed a main effect of Measures (*p* = 0.001, ${ƞ}_{p}^{2}$ = 0.113), but no Measures * Groups interaction (*p* = 0.647, ${ƞ}_{p}^{2}$ = 0.004). This shows that CSP duration similarly lengthened from Pre to Post for both the Headings (6.4 ± 7.5%) and Control groups (4.6 ± 2.6%), which also did not differ. See Table 1 for details on descriptive statistics. The results also revealed no other effects or interactions (all *p* > 0.117, all ${ƞ}_{p}^{2}$ < 0.042). Overall, this analysis shows that heading footballs increased CSP duration, but not more so than in a control group that did not head the ball. It also shows that CSP duration did not differ between the different Contraction Levels.

**Table 1:** Descriptive statistics of the CSP and CSE results.

	Pre Measures		Post Measures		Pre vs Post Measures	
	Headings	Control	Headings	Control	Headings	Control
Normalised CSP duration (%)	100.0 (0.0)	100.0 (0.0)	106.4 (7.5)	104.6 (2.6)	6.4 (7.5)	4.6 (2.6)
CSP duration (ms)	157.3 (13.1)	154.1 (14.7)	164.1 (13.7)	160.8 (16.2)	6.8 (10.8)	6.7 (3.9)
CSE (MEP amplitude; mV)	5.3 (0.7)	5.4 (0.5)	5.6 (0.6)	5.4 (0.6)	0.3 (0.3)	0.0 (0.2)

The descriptive statistics presented in Table 1 represent the mean (± 95% CIs).

To confirm that these results were not a by-product of normalising the CSP duration data, the same analysis was conducted on the non-normalised CSP duration data (in ms; Fig 2C and 2D). The results also revealed a main effect of Measures (*p* = 0.025, ${ƞ}_{p}^{2}$ = 0.084) and no Measures * Groups interaction (*p* = 0.985, ${ƞ}_{p}^{2}$ < 0.001). This analysis shows that CSP duration similarly lengthened for both groups from Pre to Post (Headings: 6.8 ± 10.8ms; Control: 6.7 ± 3.9ms). Also, both groups did not differ at Pre (*p* = 0.758, Cohen’s d = 0.079; see Table 1), suggesting that both groups had homogenous baseline CSP duration. Finally, the results revealed no other effects or interactions (all *p* > 0.108, all ${ƞ}_{p}^{2}$ < 0.044), confirming that the above results are not a by-product of normalising the CSP duration data.

### Corticospinal excitability did not differ between the Headings and Control groups

Concerning the CSE data (not shown in Fig 2), the results selectively revealed a Measures * Contraction Levels interaction (*p* = 0.011, ${ƞ}_{p}^{2}$ = 0.106). A breakdown of this interaction showed that MEP amplitude increased from Pre to Post Measure for the 40% MVC condition (*p* = 0.003, Cohen’s dz = 0.424) but not for the 80% MVC one (*p* = 0.786, Cohen’s dz = 0.035). Overall, the results of this interaction suggest that MEP amplitude increased selectively in the 40% MVC condition in response to the intervention, but only to a small extent (Cohen’s dz of 0.424) and irrespective of whether headings were performed.

More importantly, the results from the same analysis also revealed no other effects of interactions (all *p* > 0.114, all ${ƞ}_{p}^{2}$ < 0.042), suggesting that the intervention did not alter CSE. It also suggests that CSE did not differ between groups. See Table 1 for details on descriptive statistics. Overall, these results confirm that MEP amplitude did not differ between groups and was not affected by performing headings (or not).

### Increased headaches and dizziness symptoms after heading footballs

Overall, the RPQ data showed that heading footballs significantly increased headaches and dizziness symptoms as compared to the control group, providing post-hoc support to using the RPQ to assess some of the symptoms associated with sub-concussive head impacts. Whether the participants’ level of expertise in playing football mediates the headaches and dizziness symptoms could not be addressed in this work. See Table 2 for a complete report of the group questionnaire data.

**Table 2:** Group’s characteristics and RPQ results.

	**Headings**		**Control**	
Participant’s age (years old)	20.9 (1.1)		20.8 (0.9)	
Self-reported gender	25 males 5 females		25 males 5 females	
	**Pre**	**Post**	**Pre**	**Post**
Headaches	0.033	0.767	0.100	0.033
	(0.065)	(0.292)	(0.109)	(0.065)
Feeling of dizziness	0.000	0.300	0.000	0.033
	(0.000)	(0.191)	(0.000)	(0.065)
Nausea/vomiting	0.000	0.100	0.000	0.000
	(0.000)	(0.144)	(0.000)	(0.000)
Noise sensitivity	0.000	0.033	0.033	0.000
	(0.000)	(0.065)	(0.065)	(0.000)
Sleep disturbance	0.167	0.133	0.133	0.100
	(0.165)	(0.155)	(0.155)	(0.144)
Fatigue, tiring more easily	0.167	0.200	0.133	0.100
	(0.165)	(0.146)	(0.155)	(0.144)
Irritable, easily angered	0.033	0.033	0.000	0.000
	(0.065)	(0.065)	(0.000)	(0.000)
Depressed or tearful	0.067	0.033	0.100	0.067
	(0.091)	(0.065)	(0.196)	(0.131)
Frustrated or impatient	0.033	0.033	0.100	0.033
	(0.065)	(0.065)	(0.109)	(0.065)
Forgetfulness, poor memory	0.067	0.067	0.033	0.033
	(0.091)	(0.091)	(0.065)	(0.065)
Poor concentration	0.100	0.233	0.200	0.133
	(0.109)	(0.180)	(0.173)	(0.155)
Taking longer to think	0.067	0.200	0.033	0.000
	(0.091)	(0.173)	(0.065)	(0.000)
Blurred vision	0.000	0.033	0.000	0.000
	(0.000)	(0.065)	(0.000)	(0.000)
Light sensitivity	0.033	0.033	0.000	0.000
	(0.065)	(0.065)	(0.000)	(0.000)
Double vision	0.000	0.000	0.000	0.000
	(0.000)	(0.000)	(0.000)	(0.000)
Restlessness	0.000	0.067	0.100	0.100
	(0.000)	(0.131)	(0.109)	(0.109)

The descriptive statistics presented in Table 2 represent the mean (± 95% CIs).

Concerning the headaches symptoms (Fig 2E), the results revealed a Measures * Groups interaction (*p* < 0.001, ${ƞ}_{p}^{2}$ = 0.288), which further revealed that headaches symptoms increased from Pre to Post for the Headings group (*p* = 0.001, Cohen’s dz = 0.845), but not for the Control group (*p* = 0.346, Cohen’s dz = 0.263). Moreover, the Headings and Control groups did not differ at Pre (*p* = 0.313, Cohen’s dz = 0.265), but the Headings showed greater headaches symptoms than the Control group at Post (*p* < 0.001, Cohen’s d = 1.239). This analysis suggests that participants experienced headaches symptoms when heading footballs but not when controlling the ball with their lower limb.

Concerning the dizziness symptoms (Fig 2F), the results also revealed a Measures * Groups interaction (*p* = 0.012, ${ƞ}_{p}^{2}$ = 0.103), which further revealed that dizziness symptoms increased from Pre to Post for the Headings (*p* = 0.017, Cohen’s dz = 0.561), but not for the Control group (*p* = 1.000, Cohen’s d = 0.183). The Headings and Control groups did not differ at Pre (*p* = 1.000, Cohen’s d = 0.000), but the Headings showed greater dizziness symptoms than the Control group at Post (*p* = 0.012; Cohen’s d = 0.667). This result suggests that participants experienced dizziness symptoms when heading footballs but not when controlling the ball with their dominant lower limb.

Concerning the remaining RPQ-measured symptoms (Table 2), the results selectively revealed an effect of Groups (*p* = 0.041, ${ƞ}_{p}^{2}$ = 0.070) and a marginal Measures * Groups interaction (*p* = 0.096, ${ƞ}_{p}^{2}$ = 0.047) for “Taking longer to think” symptoms. Whilst the effect of Groups revealed that participants in the Headings group “took longer to think” than in the Control group (*p* = 0.041, Cohen’s d = 0.540), the marginal Measures * Groups suggests that this difference was apparent at Post (*p* = 0.043, Cohen’s d = 0.584) but not at Pre (*p* = 0.570, Cohen’s d = 0.151). Analyses of all the other symptoms revealed no effect of Measures (all *p* > 0.163, all ${ƞ}_{p}^{2}$ < 0.033), no effect of Groups (all *p* > 0.179, all ${ƞ}_{p}^{2}$ < 0.031), and no Measures * Groups interaction (all *p* > 0.112, all ${ƞ}_{p}^{2}$ < 0.043). In addition to the headaches and dizziness symptoms, this suggests that heading footballs made participants “take longer to think” as compared to controlling the ball with their lower limb. Finally, these results confirm the absence of meaningful differences within and between groups in the remaining items of the RPQ.

### Greater head acceleration upon performing football headings

Concerning the head accelerometer data (not shown in Fig 2), the results revealed greater head acceleration upon the football throws in the Headings (12.5 ± 1.9g) than in the Control subgroups (5.5 ± 1.3g; *p* < 0.001, Cohen’s d = 2.602). Whilst this difference is likely attributable to the head impacts from performing football headings, it should be noted that the Control group also showed considerable head acceleration without experiencing head impacts. This suggests that the head impacts are unlikely to fully account for the head acceleration values observed in the Headings group. Nonetheless, since the same methods were used for every participant of both groups, these results suggest that the football headings caused greater head acceleration in all participants from the Headings (n = 30) as compared to the Control group (n = 30). Whether this suggestion would be limited to practised or unpractised participants remains to be ascertained.

## Discussion

This work tested the hypothesis that the CSP duration would lengthen in response to performing football headings *and* as compared to a control group, suggesting that head impacts acutely alter brain excitability. However, the results do not support this hypothesis. Namely, the results revealed that CSP duration similarly lengthened in both the Headings and Control groups. This suggests that head impacts were not the cause of the present increases in CSP duration, as the Control group–which did not experience head impacts–also showed a comparable increase in CSP duration. Moreover, the results also revealed that MEP amplitudes of the CSP trials did not differ between groups, suggesting that head impacts did not alter CSE. However, secondary results revealed performing headings increased headaches and dizziness symptoms and resulted in greater head acceleration upon each football throw as compared to the control intervention, suggesting that head impacts were successfully induced in the Headings group only. Overall, one implication is that sub-concussive head impacts do not acutely alter brain excitability, as assessed by changes in CSP duration and CSE.

### CSP duration similarly lengthened in both groups

The main result of this work is that the CSP duration lengthened similarly for both the Headings and Control groups, suggesting that acute head impacts are not the cause of the previously reported increases in corticospinal inhibition [24]. An alternative possibility is that it is challenging to replicate the finding that performing headings–but not a control intervention in which no head impacts occur–increases CSP duration. Nevertheless, a secondary result is that MEP amplitude did not differ between groups and was not acutely modulated by performing headings or not, suggesting that acute head impacts also did not alter CSE. This finding aligns with Di Virgilio et al. (2016; ref [24]), which also found that head impacts did not alter CSE. Interestingly, CSP duration was not affected by the strength of the muscle contraction performed by the participant (80% MVC vs 40% MVC). This aligns with previous work showing that different levels of voluntary muscle contraction do not affect CSP durations [41]. Here, despite targeting a hand muscle (FDI) whereas Di Virgilio et al. (2016; ref [24]) targeted a leg muscle (rectus femoris), the present results of the Headings group closely align with the CSP lengthening reported by the same research group [24]. However, the lack of difference between the Headings and Control groups suggests that CSP lengthening–at least as measured in this study–cannot be attributed to performing football headings *per se*. Therefore, a factor unrelated to head impacts–but present in both the Headings and Control groups–must account for the present CSP lengthening or obscure the differences between the groups.

The Control group was designed to match the exercise levels–albeit low in absolute terms–of the Headings group. Thus, one possibility is that performing comparable exercise levels increased CSP duration similarly for both groups. Namely, high and low-intensity aerobic exercise alone can reduce intracortical inhibition in M1 [46, 47] but can also increase GABA concentrations in cortical motor areas [48]. Although this evidence makes it unclear if exercise should shorten or lengthen CSP duration, it nonetheless suggests that groups performing similar exercise levels would show similar changes in CSP duration. In opposition, Di Virgilio et al. (2019; ref [25]) reported that three 3-min sparring bouts increased CSP duration as compared to mock-sparring, suggesting that head impacts should increase CSP duration even if exercise levels are matched. However, Di Virgilio et al. (2016; ref [24]) did not control for exercise levels, making it unclear if the increased CSP duration they reported was due to heading footballs or to the exercise levels achieved during the intervention. Nonetheless, one obstacle for future studies will be to isolate the effects of exercise from those of sub-concussive head impacts, as the two usually co-occur and can therefore confound each other [49]. Overall, one possibility is that raising the exercise levels above the sedentary state CSP durations are typically recorded in–alone–accounts for the similar CSP lengthening in both the Headings and Control groups.

Another possibility is that changes in CSP duration between different groups can only be observed–or enhanced–when groups have different histories of concussions (for a review, see ref [22]). For instance, De Beaumont et al. (2007; ref [50]) assessed CSP duration at rest in varsity athletes with a concussion history (between 2 to 5 concussions, which occurred more than 9 months before testing) and in control participants with no concussion history. Their results showed greater CSP duration in the varsity athletes as compared to the control participants, suggesting that having a concussion history could predispose to CSP duration lengthening in response to sub-concussive head impacts (see ref [26]). De Beaumont et al. (2007; ref [50]) also reported that concussion severity positively correlated with CSP duration whereas the number of experienced concussions did not, suggesting that a history of severe concussions is an important confounding factor. Here, the participants all reported having no history of concussion in the 5 years before they participated in this study, which was shown to be sufficient to restore levels of excitation and inhibition in M1 [28]. As a result, one possibility is that the present lack of difference in CSP duration stems from a similar lack of concussion history between the Headings and Control groups.

### Increased headaches and dizziness symptoms after heading footballs

One secondary result of this work is that performing football headings increased subjective headaches and dizziness symptoms as compared to controlling the ball with the dominant lower limb, suggesting that the present methods induced head impacts in the Headings group only. Whether this result would extend to participants with (practised) and without football expertise (unpractised) remains to be ascertained. Nevertheless, these results also suggest that sub-concussive head impacts generate acute symptoms that can be readily measured in various settings (see ref [51]). Interestingly, the symptom “taking longer to think” was also marginally increased following the head impacts as compared to control, aligning with previous work suggesting that football headings deteriorate cognitive performance [10, 24, 52]. Overall, these results suggest that head impacts were experienced in the Headings group only and that the RPQ can be used to assess some of the acute subjective symptoms of sub-concussive head impacts.

### Head acceleration was greater when performing the football headings

Another secondary result is that head acceleration upon the football throws was greater when performing headings (~12.5g) as compared to the control intervention (~5.5g), suggesting that the Headings group experienced head impacts but not the Control one. However, this interpretation comes with at least three caveats. First, the extent to which the presence of head impacts can be confirmed based on accelerometer data–alone–remains unclear. A hint to this comes from the presence of head acceleration–without direct head impacts–in the control intervention (~5.5g), presumable because controlling the football with the lower limb also causes head movements. However, this result suggests that acceleration values associated with head impacts are likely to be overestimated because they co-occur with head movements, which a tri-axial accelerometer cannot disentangle. To address this, future studies should combine a tri-axial accelerometer with head surface pressure sensors [53, 54]. Second, it remains unclear if brain injuries can also be induced by abrupt head acceleration changes alone [32–34]. For instance, Varney and Varney (1995; ref [34]) simulated automobile collisions and estimated that brain acceleration changes ranging between 30 to 200g –without direct head impacts–can result in brain injury, suggesting that head impacts are not required to induce sub-concussive (or concussive) head injuries. Here, the head acceleration values of the Control group were presumably insufficient to have caused a brain injury in the absence of head impacts. As such, this explanation unlikely accounts for the similar CSP duration lengthening between the Headings and Control groups. To confirm this interpretation, future studies should include a control group that also does not experience any head acceleration by remaining at rest. Third, although the head acceleration values from the Headings group align with previous work [24, 31], whether the head acceleration values from both the Headings and Control groups would differ based on the level of football expertise remains to be ascertained.

### Limitations

Here, the participants were practised and unpractised young healthy adults. It was reasoned that the acute effects of headings on brain excitability should manifest regardless of an individual’s expertise so that football headings can be employed as a valid experimental model in humans, including experienced and non-experienced football players as well as non-football players. Whether an individual’s football expertise [55], neck strength [56] and heading technique [57], or head impact location [56], would alter the present results remains a query for future studies. Moreover, the RPQ was administered to assess the presence of subjective symptoms resulting from the sub-concussive head impacts and was not used to evaluate cognitive functions. Whether participants with expertise playing football would show lesser headaches and dizziness symptoms than those with little to no expertise remains to be addressed. Moreover, future studies should investigate how the subjective symptoms of sub-concussive head impacts interact with their associated cognitive function impairments (as in Di Virgilio et al., 2016; ref [24]), increased wet pro-inflammatory biomarker expression (see ref [58]), and brain structural insults [4, 5]. Moreover, in this study, the CSP, CSE, and RPQ results were measured acutely (i.e., before and immediately after performing headings). Whether the present results can generalise to contexts where head impacts are experienced repeatedly [10] or outside of a sporting context (i.e, falls and automobile collisions) remains to be ascertained. Furthermore, the CSP and CSE data were measured during an isometric hand muscle contraction, making it unclear if the present TMS results can generalise to contexts where brain excitability is dynamically modulated by performing a task (i.e., whilst preparing movements; see ref [59]. Whether the present changes in CSP duration stem from cortical, subcortical, or spinal structures [60] also remains to be elucidated. Finally, future replication studies should include more than 10 CSE trials to ascertain that head impacts do not alter CSE [61, 62] and record accelerometer data for all the participants of the Headings and Control groups. Whether practised and unpractised participants would show similar head acceleration in the Headings and Control groups also remains unclear.

## Conclusion

The results showed that heading footballs did not alter CSP duration differently than a control intervention that did not induce head impacts, suggesting that head impacts did not alter brain excitability. One possibility is that factors other than head impacts can confound the assessment of brain excitability when performing headings, prompting future studies to investigate what such confounding factors might be. Finally, the RPQ and accelerometer data suggest that the present methods can be used as a randomised controlled human experimental model to evaluate the effects of sub-concussive head impacts on brain functions.

## Supporting information

S1 Dataset (XLSX)
